# Effect of Potassium Iodide, Bleaching, and Microabrasion on the Colour of Silver Diamine Fluoride Stained Remineralised Caries Lesions

**DOI:** 10.3290/j.ohpd.c_2107

**Published:** 2025-07-04

**Authors:** Sarah S. Al-Angari

**Affiliations:** a Sarah S. Al-Angari Associate professor/Consultant, Department of Restorative Dental Science, College of Dentistry, King Saud University, Kingdom of Saudi Arabia. Proposal, laboratory work, collected the data, and wrote the manuscript.

**Keywords:** arrested caries, bleaching, carbamide peroxide, microabrasion, silver diamine fluoride

## Abstract

**Purpose:**

This study aimed to investigate the effect of potassium iodide (KI), bleaching, and microabrasion on the colour of caries-like lesions treated with silver diamine fluoride (SDF).

**Materials and Methods:**

Enamel specimens were demineralised and then randomised into six groups (n = 15): G1, demineralised; G2, remineralised with 38% SDF to create stained remineralised caries-like lesions (s-RCLs); G3, s-RCLs + KI; G4, s-RCLs + KI + at-home bleaching protocol (15% carbamide peroxide (CP), 4 h/d×7); G5, s-RCLs + KI + microabrasion (6.6% hydrochloric acid); G6, s-RCLs + KI + microabrasion and at-home bleaching protocol. Colour was measured spectrophotometrically at baseline, after demineralisation, and after the treatments. Outcomes were analysed using ANOVA followed by Tukey’s test (α = 0.05).

**Results:**

The increased colour change after demineralisation (ΔE ≥ 6.7) indicated the creation of white spot lesions. G2 (ΔE = 12.3) and G3 (ΔE = 11.1) were statistically significant discoloured (P ≤ 0.0039), with no statistically significant difference between them, and statistically significant darker (P <0.0001) than G1 (ΔE = 0.4) and G5 (ΔE = 4.4). G2 was statistically significantly(P ≤ 0.0325) darker than G1, G4, G5, and G6 (ΔE = 0.4, 8.1, 4.4, 7.9), respectively.

**Conclusion:**

While KI did not reduce SDF staining, microabrasion offered limited aesthetic improvement. However, 15% CP demonstrated greater efficacy in partially improving the colour outcome of SDF-stained lesions.

Aesthetic dental treatments have been highly prevalent in clinical practice in the last decades.^1^ The improvements in dental materials and conservative therapeutic approaches have directed the clinician’s attention to minimally invasive treatment strategies. Preventive measures can arrest active caries lesions, including remineralising materials such as sodium fluoride, silver diamine fluoride, casein phosphor-peptides, and bio-glasses.^23^ Silver diamine fluoride (SDF), with its commonly used concentration of 38% (25% silver, 8% ammonia, and 5% fluoride), is a topical agent applied to arrest or prevent dental caries in an effective, safe, and fast manner where silver acts as an antimicrobial agent while fluoride enhances remineralisation.^11,13,20^ Studies have demonstrated that it is superior to sodium fluoride in increasing the mineral density of demineralised enamel, and their remineralised surfaces are less susceptible to future demineralisation.^6^ However, one major drawback of SDF is the blackish discolouration associated with the remineralised arrested caries lesion. Such stains are caused by surface precipitation of silver phosphate, resulting in an unacceptable aesthetic outcome.^7,27^

The standard aesthetic management of stained arrested caries lesions usually involves surgical intervention,^7,5,27,^ which may result in unnecessary loss of highly mineralised tooth structure (non-caries lesion) for the sake of aesthetics. A suggested conservative approach to minimise its discolouration is to apply a saturated solution of potassium iodide (KI) immediately after SDF application, bleaching, and microabrasion.^6,7,12,16^ The KI is a simple approach with the potential for remineralisation and stain reduction. The iodide ions from KI react with the silver ions from SDF to form silver iodide, a pale white precipitate, instead of the dark silver oxide.^12,16^ This transformation minimises the immediate visual impact of the staining. Nevertheless, concerns regarding its long-term colour stability persist and require further investigation. The subsequent application of bleaching agents (eg, carbamide peroxide) and/or microabrasion techniques may address residual discolouration and improve the overall aesthetic appearance. Bleaching is a fast, effective method to oxidise chromophore molecules, while microabrasion physically removes the superficial stained layer, potentially containing residual silver compounds.^2^ However, they may result in dentine hypersensitivity, soft tissue irritation and affect the shear bonding strength of restorative materials placed on the treated surfaces.

To the best of our knowledge, no previous studies have evaluated the sequential application of KI, followed by bleaching and/or microabrasion, to optimise aesthetic outcomes by chemically altering the staining mechanism and physically removing affected enamel while maintaining a conservative treatment approach. The aim of this study was to investigate the efficacy of different aesthetic approaches (KI, at-home bleaching, microabrasion, and a their combination) on the colour of artificially created caries-like lesions treated with SDF.

## MATERIALS AND METHODS

### Experimental Design

Artificial caries-like lesions were created on human enamel, then remineralised using SDF to create stained remineralised caries-like lesions (s-RCLs). The study investigated the aesthetic colour outcome of s-RCLs at six levels: G1: negative control (demineralised/no treatment); G2: positive control (s-RCLs); G3: s-RCLs + potassium iodide (KI); G4: s-RCLs +KI + at-home bleaching; G5: s-RCLs +KI+ microabrasion; G6: s-RCLs+ KI+ + microabrasion + at-home bleaching. The study outcome was colour change (ΔE) measured at three time points: baseline, after demineralisation, and after the designated aesthetic treatment, performed using an optical spectrophotometer.

### Specimen Preparation

The experimental units were enamel slabs (4 × 4 × 2 mm) from human molars, sound and free of cracks or defects. Ninety specimens were chosen after the approval by the King Saud University institutional review board (IRB# E-19-3836), then sectioned from the buccal and lingual area using a low-speed diamond saw (Isomet, Buehler, Lake Bluff, IL, USA) then embedded in acrylic resin blocks (10 × 10 × 8 mm; Varidur, Buehler, Lake Bluff, IL, USA). They were sequentially ground flat using 500, 1200, 2400, and 4000 silicon carbide grinding papers (MD Fuga, Struers, Cleveland, OH, USA), polished with 1-µm diamond suspension (Struers, Cleveland, OH, USA), then sonicated in a detergent solution (Micro-90, International Products Corporation, Burlington, USA). After polishing, the specimens were placed under running deionised water for 3 min, then stored in a refrigerator at approximately 100% relative humidity at 4ºC (Kenmore; Whirlpool, Benton Harbor, MI, USA) until the initiation of the experiment.

### Caries-like Lesion Creation

Enamel caries-like lesions were initiated by placing the specimens in a demineralising solution, as described by Lippert et al (2011).^18^ All specimens were demineralised for seven days in a solution containing 0.1 M lactic acid, 4.1 mM Ca (as CaCl_2_. 2H_2_O), 8.0 mM PO_4_ (as KH_2_PO_4_), and 1.0% w/v carboxymethylcellulose (Sigma-Aldrich, St. Louis, MO, USA) with pH adjusted to 5.0 using KOH at 37°C. The demineralising agent was not stirred or replaced throughout the demineralisation period.

### Staining and Treatment

After demineralisation, specimens were randomly divided, using a computer-based software system, into six major groups (n = 15) based on the treatment protocol (Table 1). Group 1 (negative control): demineralised/no treatment. Group 2 (positive control); treated with 38% SDF (SDF; Advantage Arrest, Elevate Oral Care LLC, West Palm Beach, FL, USA) for 2 min^12^ to create s-RCLs. Group 3: s-RCLs treated immediately with saturated KI solution. Group 4, s-RCLs treated with KI solution followed by at-home bleaching protocol, using 15% carbamide peroxide. Group 5, s-RCLs, treated with KI solution followed by microabrasion (using 6.6% hydrochloric acid/pumice). Group 6, s-RCLs, treated with KI solution followed by microabrasion, then at-home bleaching protocol.

**Table 1:** Group definitions based on the treatment protocol used

Groups	Treatment protocol
G1	Negative control (demineralisation/no treatment)
G2	Positive control (s-RCL treated with 38% SDF)
G3	s-RCL + KI
G4	s-RCL + KI + at-home bleaching
G5	s-RCL + KI + microabrasion
G6	s-RCL + KI + microabrasion + at-home bleaching
s-RCL = stained remineralised caries-like lesion; SDF = silver diamine fluoride; KI = potassium iodide solution, at-home bleaching; 15% carbamide peroxide bleaching gel, microabrasion = 6.6% hydrochloric acid/pumice.

### KI Preparation and Application

The KI solution (2.36 mol/L) was prepared in the laboratory using distilled water and KI powder (SIGMA-ALDRICH Co., St. Louis, USA). The creamy white solution was brushed on the surface of specimens in G3, G4, G5, and G6, then removed when the solution’s reaction products turned clear. It was washed off with copious distilled water, and then the surface was air-dried.

### Dental Bleaching Efficacy

Dental bleaching was performed on groups 4 and 6; they were treated with an at-home bleaching protocol, 15% carbamide peroxide (CP) (pH 6.5; Opalescence PF, Ultradent Products, South Jordan, UT, USA). The bleaching gel was applied onto the top enamel surface (0.5–1.0 mm thick) and kept for 4 h each day in an incubator at 37°C for a total treatment of seven days.^4,7^ After bleaching, specimens were rinsed with running distilled water for 1 min to remove the bleaching agent’s residue, blot-dried, and stored in a moist environment at 4°C for colour measurements, performed immediately afterwards.

### Dental Microabrasion Test

It was performed on groups 5 and 6 (prior to bleaching).^10,24^ The microabrasive product (6.6% hydrochloric acid, Opaluster, Ultradent Products, South Jordan, UT, USA), was applied on each specimen (1.0 mm thick), using a prophylaxis rubber cup to rub the material on the surface for one min., then specimens were rinsed and dried. Specimens were then polished with a fluoridated prophylaxis paste (Vigodent Coltene SA Indústria e Comércio; Rio de Janeiro, RJ, Brasil) and then a 2% neutral-Ph sodium fluoride gel was applied for 4 min as per the manufacturer’s instruction.^24^

### Colour Assessment

L*a*b* values (Commision Internationale de l’Eclairage) were taken for each specimen at baseline, immediately after demineralisation, and after the assigned treatment. Measurements were performed using a spectrophotometer (ColorEye 7000A, GretaMacBeth LLC, NY USA; light beam diameter of 0.3 mm) and repeated three times. The colour difference (ΔE) was calculated using the following equation, representing colour changes after demineralisation (ΔEDemin: demineralisation-baseline) and after the assigned treatment (ΔEtreatment: treatment-staining):

ΔE = {(ΔL*)^2^+(Δa*)^2^+(Δb*)^2^}^1/2^

### Statistical Analysis

The colour change (ΔE) was analysed using one-way ANOVA (α = 0.05), followed by a multiple comparison test (Tukey’s test). Statistical analysis was performed using SPSS software (IBM SPSS Statistics v.26, IBM, Armonk, NY, USA). Prior to the study, calculations showed that a planned sample size of 15 per group was designed to have 89% power to detect a ΔE difference (0.8) between groups, assuming a 5% significance level and standard deviation of 0.7.

## RESULTS

An increase in the mean colour change (ΔE ≥ 6.7) was observed after demineralisation, indicating successful white spot lesion formation. No statistically significant differences were found between each specimen after demineralisation (P >0.05). Groups 2 (SDF) and 3 (SDF+KI) exhibited statistically significant discolouration (P ≤ 0.0039) compared to their demineralised state, confirming the efficacy of the SDF staining process. Additionally, G5 demonstrated a statistically significant colour change relative to its demineralised state (P = 0.0224), suggesting that the treatment did not effectively improve the discolouration.

The treatment assessment among groups was in comparison to the positive control group G2. Both G2 (ΔE = 12.3) and G3 (ΔE = 11.1) had the highest statistically significant colour (black) change, with no significant difference between them, indicating that the addition of KI did not statistically significantly improve the colour outcome. However, they were statistically significantly darker (P < 0.0001) than G1 (ΔE =0.4) and G5 (ΔE = 4.4). G2 was statistically significantly darker than G1, G4, G5, and G6 (P ≤ 0.0325), while no statistically significant difference was observed between G4 and G6 (Table 2).

**Table 2:** Colour change (ΔE) means (standard deviation) after demineralisation and treatment

Groups	ΔEDemin^+^	ΔETreatment^+^
G1	7.2 (3.2)	A,a	0.4 (1.2)	B,a
G2	7.9 (3.2)	A,a	12.3 (4.7)	B,b,c
G3	6.7 (3.7)	A,a	11.1 (4.4)	B,c,b,d,f
G4	7.5 (4.2)	A,a	8.1 (4.5)	A,d,c,e,f
G5	7.3 (3.7)	A,a	4.4 (1.9)	B,e,d,f
G6	6.7 (3.9)	A,a	7.9 (3.0)	A,f,c,d,e
Uppercase letters indicate significant difference within treatment (row, p<0.05); while lower case letters indicate significant difference among treatments (column, P < 0.05). ΔEDemin+: demineralisation – baseline, and ΔEtreatment +: treatment – demineralisation. G1 = negative control (demineralised/no treatment); G2 = positive control (stained remineralised caries-like lesions s-RCLs using silver diamine fluoride); G3 = s-RCLs + potassium iodide (KI); G4 = s-RCLs + KI + at-home bleaching; G5 = s-RCLs + KI + microabrasion; G6 = s-RCLs + KI microabrasion + at-home bleaching.

## DISCUSSION

This study created an *in-vitro* model simulating remineralised caries-like lesions (s-RCLs) to standardise experimental variables such as demineralisation (mineral loss and lesion depth), staining, and remineralisation (38% SDF) protocols.^6,7^ Thirty-eight per cent (38%) SDF was selected for its rapid and effective remineralisation properties, attributed to the synergistic effect of its high concentration of fluoride (the highest applied clinically at 44,800 ppm) and silver (255,000 ppm), along with ammonia, which elevates the alkalinity (pH value of 9–10) and acts as a stabiliser.^8,26,27^ A saturated KI solution was used to investigate its effect on the colour improvement of black stains associated with SDF.

Colour was objectively quantified using the CIE L*a*b* system to eliminate human error, in which a colour difference (ΔE) equal to or exceeding 3.3 units was considered clinically perceptible.^14^ It is noteworthy to mention that all colour changes (ΔE) values, after demineralisation and treatment, exceed the established thresholds for acceptability and perceptibility.^21^ The demineralisation process successfully created white spot lesions, as evidenced by the high mean change in colour lightness (ΔE ≥ 6.7). This change was characterised by an increase in the lightness (L*) and a decrease in the greenness (a*) and blueness (b*) coordinates, directing the ΔE to lighter colour improvement.^2^ G1 showed minimal change (ΔE 0.4) following incubation; this minute change is attributed to rehydration. Additionally, the treatment of SDF in G2, serving as a baseline for comparison with other treatments, produced distinct (blackish) visual discolouration (ΔE = 12.2). The blackish discolouration is attributed to the oxidation of ionic silver to metallic silver and silver oxide, forming silver-protein and silver phosphate compounds that precipitate and cause tooth discolouration.^8^ Paradoxically, these stains signify successful remineralisation, indicating an area highly concentrated in calcium, phosphorus, fluoride, and silver ions.^8^

The use of KI to improve discolouration in SDF surfaces remains controversial, with significant inter-study differences and inadequate clinical evidence.^15,22^ Studies reporting improved colour outcomes with KI indicated that it was time, light, and temperature-dependent.^9,17,19^ It may temporarily improve the staining at the initial treatment stage by reacting with the free silver ions, resulting in a creamy white precipitate of silver iodide.^9,17,19^ However, with time, exposure to light, and increased temperature (eg, in an incubator or oral environment), it accelerates the breakdown of silver iodide (photosensitive structure) into silver and iodine, leading to a darkening of the treated area, even under a restoration.^9^ These findings align with our study results, as the colour outcome in G3 (SDF+KI) produced less dark stains (ΔE= 11.1) than G2, yet were not statistically significantly different. Furthermore, all groups demonstrated statistically significantly lower colour values than G2, except for G3. The initial effect of KI may have slightly improved the SDF colour in G3, which justifies why it was slightly lower than G2 and did notstatistically significantly differ from the other groups. The laboratory conditions of this study allowed for specimens to be kept in an incubator (37°C) to mimic the intraoral conditions and to be exposed to light during the process of colour measurement, which may have accelerated the darkening effect in G3 and justified its similarity to G2. However, this minute colour improvement of KI was expected, as KI’s main use is to enhance the penetration of SDF into the tooth rather than directly affecting the colour.^17^ This is due to KI’s hygroscopic property, which increases the solubility and decreases the surface tension of SDF, thus altering the rate of silver ions formation, potentially leading to a transient improved colour outcome.^12^

The at-home bleaching protocol was chosen because of its longer exposure time (4 h daily for 1 week, totalling 28 h), which yielded superior and more consistent colour improvement results.^3,6,7^ The increased colour lightness (ΔE = 8.1) was perceptible yet could not exceed the staining values, resulting in a residual dark stain. This response was attributed to the type of stain, as metallic stains have a heavy molecular weight and are less water-soluble, thus are difficult to degrade and oxidise by peroxides, which can easily oxidise organic chromophores, which is similar to previous studies.^2,3,6,7^ Furthermore, microabrasion had half the improvement (ΔE = 4.4) compared to bleaching (ΔE = 8.1). Its chemical/mechanical process may have minimised silver deposition and discolouration limited to the outer enamel surface (25–200 μm), yet was unable to effectively penetrate embedded stains of SDF in deeper layers and lighten the colour, which supports our findings that KI enhanced the penetration of the SDF into deeper layers.^25^ Eroding superficial enamel may facilitate channels for bleaching agents to oxidise deeper stains, demonstrating statistically significant results when discolourations are non-metallic (organic).^2^ However, when combined with bleaching, it did not statistically significantly differ (ΔE = 7.9) from bleaching alone.

Although *in-vitro* studies are highly standardised, with better control over variables (lesion type, depth, stain), they cannot replicate the complex biological processes involved in s-RCL formation. Therefore, clinical extrapolation of the results should be done with caution. In summary, the findings of this study indicated that the KI solution had no statistical significantly effect on the colour improvement of SDF stains. Additionally, minimally invasive techniques such as microabrasion, alone or combined with bleaching, demonstrated no significant effect on the colour outcome of the metallic stains associated with SDF. Therefore, the unnecessary removal of tooth substrate using microabrasion is not recommended. At-home bleaching may be used as an initial step to improve the colour outcome and minimise invasive tooth removal. However, it may result in a residual blackish discolouration. These results suggest the use of SDF with caution and recommend limiting its use to non-aesthetic regions. Further studies should be conducted to assess the potential impact of the aesthetic treatments on the enamel’s morphological structure, such as surface roughness and microhardness.

## CONCLUSION

Within the limitations of this *in-vitro* study, SDF has effectively stained demineralised lesions. However, KI’s application did not significantly differ from the SDF staining. Aesthetic approaches, such as microabrasion treatment, were shown to have a limited effect on lightening the SDF-stained lesions. At-home bleaching protocol (15% CP) offered a potentially viable option to partially remove the stains associated with SDF.

### Ethics Statement

This study received institutional review board (IRB) approval from King Saud University (#IRB E-19-3836).

### Conflicts of Interest

The author declares no conflict of interest.

### Funding

This research received no external funding.

### Data Availability

All data generated or analysed during this study are included in this published article and the data sets used during the current study are available from the corresponding author on reasonable request.

### Acknowledgements

The author would like to thank Dr Abdulaziz Al-Angari, Associate professor of Epidemiology, Department of Epidemiology and Biostatistics, for the insight and expertise in the statistical analysis that greatly assisted the research.
